# Risk Factors and Characterization of Plasmodium Vivax-Associated Admissions to Pediatric Intensive Care Units in the Brazilian Amazon

**DOI:** 10.1371/journal.pone.0035406

**Published:** 2012-04-16

**Authors:** Ellen Fátima Caetano Lança, Belisa Maria Lopes Magalhães, Sheila Vitor-Silva, André Machado Siqueira, Silvana Gomes Benzecry, Márcia Almeida Araújo Alexandre, Connor O'Brien, Quique Bassat, Marcus Vinícius Guimarães Lacerda

**Affiliations:** 1 Universidade do Estado do Amazonas, Manaus, Amazonas, Brazil; 2 Fundação de Medicina Tropical Dr. Heitor Vieira Dourado, Manaus, Amazonas, Brazil; 3 Universidade Nilton Lins, Manaus, Amazonas, Brazil; 4 National Institute of Science and Technology for Innovation in Neglected Diseases, Rio de Janeiro, Brazil; 5 Universidade Federal do Amazonas, Manaus, Amazonas, Brazil; 6 Columbia University Medical Center, New York, New York, United States of America; 7 Centre de Recerca en Salut Internacional de Barcelona (CRESIB)/Hospital Clínic, Universitat de Barcelona, Barcelona, Spain; Institut Pasteur, France

## Abstract

**Background:**

*Plasmodium vivax* is responsible for a significant proportion of malaria cases worldwide and is increasingly reported as a cause of severe disease. The objective of this study was to characterize severe vivax disease among children hospitalized in intensive care units (ICUs) in the Western Brazilian Amazon, and to identify risk factors associated with disease severity.

**Methods and Findings:**

In this retrospective study, clinical records of 34 children, 0–14 years of age hospitalized in the 11 public pediatric and neonatal ICUs of the Manaus area, were reviewed. *P. falciparum* monoinfection or *P. falciparum/P. vivax* mixed infection was diagnosed by microscopy in 10 cases, while *P. vivax* monoinfection was confirmed in the remaining 24 cases. Two of the 24 patients with *P. vivax* monoinfection died. Respiratory distress, shock and severe anemia were the most frequent complications associated with *P. vivax* infection. Ninety-one children hospitalized with *P. vivax* monoinfections but not requiring ICU were consecutively recruited in a tertiary care hospital for infectious diseases to serve as a reference population (comparators). Male sex (p = 0.039), age less than five years (p = 0.028), parasitemia greater than 500/mm^3^ (p = 0.018), and the presence of any acute (p = 0.023) or chronic (p = 0.017) co-morbidity were independently associated with ICU admission. At least one of the WHO severity criteria for malaria (formerly validated for *P. falciparum*) was present in 23/24 (95.8%) of the patients admitted to the ICU and in 17/91 (18.7%) of controls, making these criteria a good predictor of ICU admission (p = 0.001). The only investigated criterion not associated with ICU admission was hyperbilirubinemia (p = 0.513)].

**Conclusions:**

Our study points to the importance of *P. vivax*-associated severe disease in children, causing 72.5% of the malaria admissions to pediatric ICUs. WHO severity criteria demonstrated good sensitivity in predicting severe *P. vivax* infection in this small case series.

## Introduction

Malaria is one of the most important public health problems in the world, with almost half of the world's population at risk of disease and an estimated 800.000 deaths annually, mainly in children under five years of age [1]. Of the various *Plasmodium* species affecting humans, *Plasmodium vivax* was previously considered a relatively benign infection. Recent reports, however, have linked *P. vivax* to severe disease [2], [3].

Malaria in Brazil has been by and large attributed to *P. vivax* infection since the 1990's [4]. In 2009, Brazil reported 308,498 cases of malaria (257,571 of *P. vivax*), predominantly in the Amazon Region, representing 54.9% of all the malaria reported in the Americas [1]. In the last two decades, the city of Manaus (capital of the Amazonas State) has faced successive outbreaks of malaria due to intense immigration, deforestation and unplanned human settlements [5]. Today, with an estimated population of 2 million people, Manaus is one of three cities, which account for 25% of all reported malaria cases in Brazil. High case burdens persist despite a well-structured public health system with universal access to diagnosis (based on thick blood smear) and treatment.

Previously, *P. vivax* was considered a non-fatal infection. This perception, however, has changed in recent years, and vivax has become recognized as a cause of severe malarial disease. Severe clinical complications of *P. vivax* infection have been reported in Brazil for the past decade, especially in Manaus [6]–[12]. Despite being a significant source of morbidity, to date, no data on risk factors of severe *P. vivax* disease have been published in Latin America. The current data on predictors of clinical outcomes originates from regions where *P. falciparum* and *P. vivax* are equally prevalent, which could make the clinical tools inappropriate for use in managing vivax monoinfections [13]. This lack of prioritizing predictors of severity in *P. vivax* is due to the fact that fatalities related to this species remain uncommon. According to the official statistics, between 1998 and 2008, only 234 deaths related to *P. vivax* infection were reported in the Brazilian Amazon [4].

The first attempt to assemble the available knowledge on severe malaria was conducted at an informal technical meeting convened by the World Health Organization (WHO) in Kuala Lumpur, Malaysia in 1985 [14]. The document focused on *P. falciparum* infection, since, at that time, *P. vivax* was not thought to cause severe disease. Throughout the years, no effort has been made to validate or standardize specific severity criteria for vivax infections, and current definitions seem to be heterogeneous. This absence of consistency poses technical problems for literature review, meta-analyses and estimation of risk factors.

Alarmingly, the incidence of severe infection appears to be increasing [15]. Data from Manaus clearly demonstrate this increase in a setting where uniform study criteria are applied across time. The mechanism behind this increase remains poorly understood as are the clinical predictors of severity and outcome. The most robust endpoint for validating a criterion linked to severity is death, but in vivax infections in Brazil the number of deaths is small, which limits prognostic studies. Given this limitation a surrogate is required to replace death. Intensive care unit (ICU) admission could serve as this surrogate marker of severity, in combination with well-validated severity scoring systems that predict mortality in ICU patients. A uniform comparator is urgently needed to help establish management guidelines for vivax infections, especially for children under the age of 14, which accounted for 24.3% of all malaria infections in 2010 in Manaus (SIVEP, Malaria 2010).

This study aimed to characterize all children under 14 years of age admitted with a malaria diagnosis to an ICU in the Manaus area from 2004 to 2009, to assess potential risk factors associated with ICU admission (as proxy of clinical severity), and secondarily to address the applicability of WHO falciparum severity criteria to vivax infection, by comparing these cases to *P. vivax* hospitalized children not requiring intensive care.

## Methods

### Ethical considerations

The review of clinical files was approved by the Ethics Review Board (ERB) of the *Fundação de Medicina Tropical Dr. Heitor Vieira Dourado* (approval number 1980), as well as by the ERB of the Health Secretariat of Manaus and the Health Secretariat of the Amazonas State. All data analyzed were anonymized. Since data were obtained exclusively from clinical charts, the ERB gave a waiver of informed consent.

### Study Sites and Patient Selection

The selection of cases was performed in the city of Manaus, capital of the Amazonas State, where *Anopheles darlingi* is the major malaria vector and the annual parasite index (API) was 11.5 cases/1,000 inhabitants, in 2009. The city has universal health assistance for citizens (*Sistema Único de Saúde - SUS*), and offers five public pediatric ICUs (44 total available beds) and six neonatal ICUs (50 total available beds) in 11 tertiary care pediatric hospitals and maternity wards. The infrastructure of all 11 hospitals is very similar and staff pediatricians are from the same cooperative society, which utilizes a single set of admission criteria and clinical management guidelines for ICU admission (severe compromise of respiratory and/or hemodynamic functions and/or coma). Since every febrile patient in Manaus is subject to a thick blood smear (TBS) and the symptoms of uncomplicated *P. vivax* and *P. falciparum* malaria are similar, treatment seeking bias seems unlikely. Since the access to tertiary care hospitals is relatively easy, we assume that all critically ill patients seeking health care were subsequently hospitalized in one of Manaus' ICUs. Patients in private ICUs in Manaus were excluded from this study because the patients are generally from non-malaria endemic areas. Patients infected with *P. vivax* that required an ICU admission were compared to children of the same age range infected with *P. vivax* not requiring an ICU admission but concurrently admitted to a tertiary care center for infectious diseases (*Fundação de Medicina Tropical Dr. Heitor Vieira Dourado*).

### Study Design

This study is a retrospective review of clinical and laboratory records from hospital databases. Case files for children 0–14 years old admitted to any of the ICUs with a suspicion of malaria between January 2004 to December 2009 were reviewed for: (1) ICU logbooks (in which demographic data from each admitted patient, a presumptive diagnosis at admission and a final diagnosis at discharge are systematically registered), (2) hospital laboratory logbooks (in which the semi-quantitative results of TBSs performed in each hospital are systematically registered), and (3) SIVEP-Malaria (the National Malaria Information System available on-line, which registers results of TBSs performed in each reporting unit such as the hospitals of interest). Cases were included if there was evidence of malaria diagnosis by microscopy in any of the three information systems searched. The study enrolled only patients after 2004 because of better reliability of the National Malaria Information System (SIVEP-Malaria) and access to the clinical files, avoiding retrieval bias.

As comparators, clinical and laboratory data from children of the same age group admitted between January 2009 and July 2010 to the *Fundação de Medicina Tropical Dr. Heitor Vieira Dourado* with a diagnosis of malaria were retrieved electronically from the computerized hospital records system (iDoctor®) and reviewed. With the exception of automated full blood count, routinely requested for all these hospitalized children, more specific laboratory tests (such as biochemistry and arterial gas analysis) were requested only if clinical symptoms were suggestive of complications. As part of routine follow-up, all malaria admitted children were reassessed in the Outpatient Clinics seven days after discharge to guarantee adequate clinical and parasitological follow-up.

### Diagnosis of Malaria and Quantification of Parasitemia

TBSs are routinely performed for the diagnosis of malaria in Brazil, prepared as recommended by the Walker technique [16] and evaluated by a local microscopist. The results of peripheral parasitemia are given using the following semi-quantitative system: ½+ (200–300 parasites/mm^3^); 1+ (301–500 parasites/mm^3^); 2+ (501–10,000 parasites/mm^3^); 3+ (10,001–100,000 parasites/mm^3^); and 4+ (>100,001 parasites/mm^3^). The cut-off to define high parasitemia (in the final analysis) was based on the distribution of semi-quantitative parasitemia results. All positive slides and 10% of negative slides are routinely reviewed in a reference unit with experienced microscopists. In case of divergence, the reviewed result is updated in the on-line system. PCR diagnosis for malaria was not routinely performed in these patients. Only children with a positive TBS for *Plasmodium* spp. are treated according to the Brazilian Anti-Malarial Treatment Guidelines, which recommends chloroquine (25 mg/kg over 3 days)+primaquine (0.5 mg/kg/day over 7 days) for *P. vivax* infection and artemether/lumefantrine for 3 days for non-severe *P. falciparum* infection. Severe *P. falciparum* infections are treated with parenteral artemether or artesunate for 7 days.

### Data collection and definitions

Data for each admitted individual was systematically retrieved from the medical charts by the same member of the study team, and included the following variables: sex, age, malarial species, semi-quantitative parasitemia, duration of disease (in days) prior to the admission to the ICU or ward, outcome, presence of a given acute co-morbidity other than malaria or a chronic disease confirmed through a reliable diagnostic method, and presence of any WHO-defined severe malaria criteria [17]. Briefly, WHO criteria for severe falciparum malaria validated for adults and children include: (1) impaired consciousness or unrousable coma (Blantyre coma scale ≤2 or Glasgow coma scale ≤10); (2) prostration, i.e. generalized weakness such that the patient is unable to walk or sit up without assistance; (3) failure to feed; (4) multiple convulsions (greater than two episodes in 24 h); (5) respiratory distress defined as the presence of deep (acidotic) breathing or retractions; (6) circulatory collapse or shock, systolic blood pressure <50 mmHg (algid malaria) and/or need for vasopressor support; (7) clinical jaundice or total bilirubin >3 mg/dL; (8) hemoglobinuria (blackwater fever); (9) abnormal spontaneous bleeding (disseminated intravascular coagulation); (10) pulmonary edema (radiological); (11) hypoglycemia (blood glucose <40 mg/dL); (12) metabolic acidosis (plasma bicarbonate <15 mmol/L); (13) severe anemia (Hb<5 g/dL); (14) hyperlactatemia (lactate >5 mmol/L); or (15) acute renal failure (serum creatinine >3 mg/dL). Acute respiratory distress syndrome (ARDS) was defined as acute bilateral lung infiltrates in chest X-rays, and a PaO_2_∶FiO_2_<200 mmHg. The data retrieved were reviewed by another member of the study team and discrepancies resolved by a third member if necessary.

In all ICUs, collection of venous blood samples for aerobic culture and arterial blood for gas analysis was routinely carried out on admission prior to administration of antibiotics. From all the patients hospitalized in the ICU, the Pediatric Index of Mortality (PIM) score was calculated with data from the first hour of admission, based on the systolic blood pressure, pupillary reactions to bright light, PaO_2_, FiO_2_, base excess in arterial blood, need for mechanical ventilation at any time during the first hour in ICU, elective admission, and presence of concomitant diagnoses [18]. Severity was defined in the presence of at least one of the aforementioned WHO severity criteria. As blood gas analysis samples were not systematically collected for patients not admitted to the ICU, hyperlactatemia and metabolic acidosis were not used to classify severity nor assessed as a risk factor associated with ICU admission. The disease was classified as being directly caused by plasmodial infection if no other cause was identified and no concurrent acute or chronic diseases were confirmed.

### Statistical Analyses

Data were analyzed using SPSS® version 16.0 for Windows (SPSS Inc.® Chicago, IL, USA). Proportions of fatality were compared by Fisher exact test (corrected by Yates' test if necessary); differences were considered statistically significant for p<0.05. The crude *Odds Ratio* (OR) with its respective 95% Confidence Interval (95% CI) was determined considering the admission to ICU as the dependent variable. Logistic regression was used for the multivariate analyses and the adjusted *Odds Ratios* with 95% CI were also calculated. A multivariate logistic regression was performed with admission to the ICU as the outcome, using an automated backward and forward stepwise estimation. All variables that were associated with admission to the ICU at a significance level of p<0.10 in the univariate analysis were included in the multivariate analysis. Statistical significance was considered if p<0.05 in the Hosmer-Lemeshow goodness-of-fit test.

## Results

**Figure 1:**
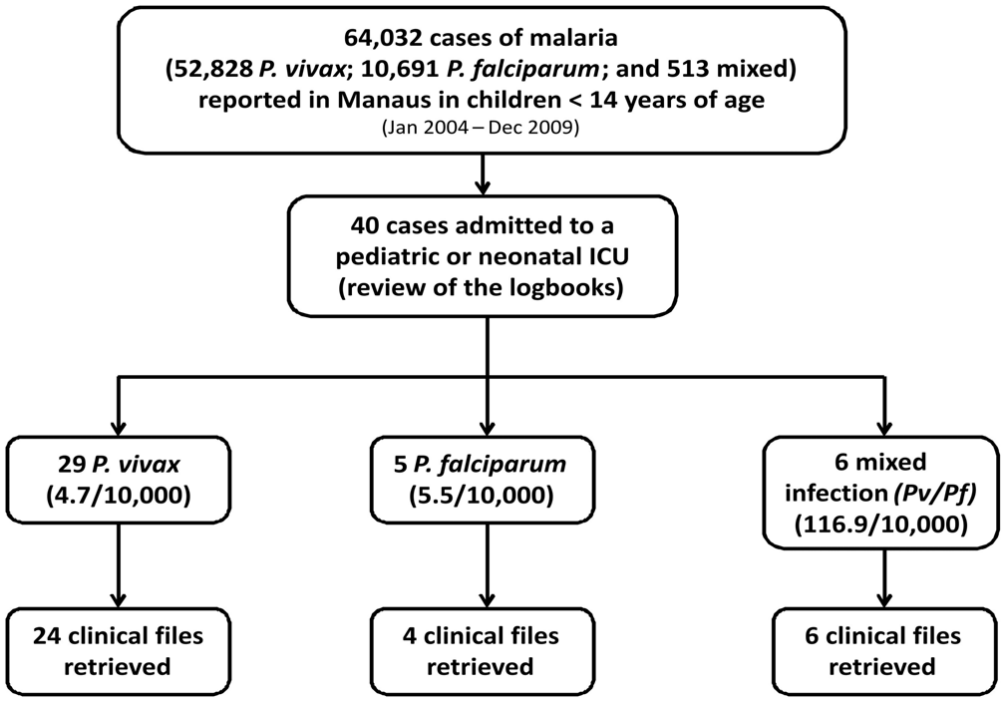
Flow diagram of patients admitted to ICUs enrolled in the analysis.

According to the official reporting systems, during the period of 2004–2009, 64,032 children 0–14 years of age were diagnosed with malaria in Manaus (52,828 with vivax; 10,691 with falciparum and 513 with both species). The review of logbooks disclosed that 40 of these children were admitted with malaria to one of the 11 pediatric or neonatal ICUs in Manaus. Thirty-four clinical files were actually retrieved from these 40 cases (29 of which were due to *P. vivax* infections), as shown in Figure 1, and six cases were excluded because records could not be found. A rough estimation of the risk of ICU admission per species showed similar relative risks for *P. vivax* (4.7/10,000 cases) and *P. falciparum* (5.5/10,000 cases), but a much higher relative risk for mixed infections (116.9/10,000 cases).

Table 1 summarizes the clinical description of the 10 ICU cases in which *P. falciparum* was diagnosed (four cases as monoinfection, and six cases of *P. falciparum/P. vivax* mixed infection). Altogether, respiratory distress (7/10) and shock (7/10) were the most common clinical complications in these children and the case fatality rate was 30.0% (3/10) (only two with *P. falciparum* monoinfection). No other concurrent cause for ICU admission was found in 40.0% (4/10) of the children while in the other six children, acute and/or chronic conditions were probably acting synergistically to produce the complications. Only one concomitant bacterial infection was diagnosed (*Pseudomonas aeruginosa*) in a child with *P. falciparum* monoinfection. In all 10 patients, at least one of the WHO criteria for severe malaria was present.

**Table 1:** Clinical information from 10 children 0–14 years of age admitted to any of the pediatric or neonatal ICUs in Manaus, from January 2004 to December 2009, with parasitological diagnosis of *P. falciparum* or mixed infection (*P. falciparum/P. vivax*).

Patient	Species	Age/gender	Time of disease (days)	Associated acute comorbidity	Associated chronic comorbidity	Respiratory distress	Severe anemia	ARF	Coma	Jaundice	Shock	Metabolic acidosis	Low glucose	PIM score (%)	Outcome
1	*P.f.*	6 y/F	14	Rotavirus gastroenteritis*+Sepsis* (*Pseudomonas aeruginosa*)	Congenital cardiopathy*	+					+			84.7	Died
2	*P.f.*	7 y/F	5			+ (ARDS)	+		+					40.0	Recovered
3	*P.f.*	10 y/F	5					+	+	+	+	+	+	45.7	Died
4	*P.f.*	1 mo/M	7			+	+							10.6	Recovered
5	*P.f./P.v.*	7 mo/F	2	Rotavirus gastroenteritis		+					+			7.4	Recovered
6	*P.f./P.v.*	13 y/M	30		G6PD deficiency*		+	+**				+		1.3	Recovered
7	*P.f./P.v.*	5 mo/F	48		Malnutrition*	+				+	+			NA	Died
8	*P.f./P.v.*	3 y/M	7	Rotavirus gastroenteritis		+ (ARDS)					+			15.4	Recovered
9	*P.f./P.v.*	9 y/M	7		Sickle cell anemia* + Malnutrition		+			+	+			1.4	Recovered
10	*P.f./P.v.*	2 mo/M	2			+ (ARDS)					+			13.9	Recovered

Y: years; mo: months; d: days; NA: Non-available; ARDS: acute respiratory distress syndrome; ARF: acute renal failure; PIM: Pediatric Index of Mortality.

* **Malnutrition** confirmed by body mass index (BMI) Z-score <−2; **rotavirus gastroenteritis** confirmed by immunochromatographic rapid test in the stool; **sepsis** confirmed by two positive blood cultures; **G6PD deficiency** confirmed by qualitative Brewer's test; **congenital cardiopathy** confirmed by echocardiogram; **sickle cell anemia** confirmed by electrophoresis; ARDS defined as acute bilateral lung infiltrates and a PaO_2_:FiO_2_<200 mmHg.

** Blackwater fever.

Clinical and laboratory data from the 24 children with *P. vivax* monoinfection admitted to the ICU are presented in tables 2 and 3, respectively. Considering all the patients (admitted or not to the ICU), 46/108 (∼42%) patients presented with semi-quantitative parasitemia ≥2+ (500–10,000 parasites/mm^3^), and therefore 500 parasites/mm^3^ (the lower bound of this range) was chosen as the cut-off to define high parasitemia in our analysis, enabling similar number of patients in both categories (low and high parasitemia). Respiratory distress (16/24) and shock (13/24) were also the most common complications associated with this species. Three out of 16 patients with respiratory distress had ARDS and 8/16 presented with simultaneous metabolic acidosis. No other apparent concurrent cause for ICU admission was found in 10/24 (41.6%) children, while in the remaining 14 children there was evidence of the participation of acute and/or chronic conditions in the occurrence of complications. All children were treated with chloroquine (through nasogastric catheter when the child was intubated), primaquine after discharge, and one or more empiric antibiotic regimens. Although, only two patients (one of which with dual infection) presented positive blood cultures during the admission (table 2). Two patients with confirmed glucose-6-phosphate dehydrogenase (G6PD) deficiency experienced severe hemolysis, hemoglobinuria and acute renal failure after the beginning of primaquine (all started with hemoglobinuria during the second or third day of treatment). All the patients who developed shock syndrome required administration of vasoactive amines. With the exception of one patient with a previous diagnosis of idiopathic thrombocytopenic purpura (ITP), who presented with moderate bleeding and was admitted to the ICU as a result, all remaining 23 patients had one or more WHO severity criteria for malaria. All the patients demonstrated a TBS negative for *Plasmodium* spp. following chloroquine treatment.

**Table 2:** Detailed clinical information from 24 children 0–14 years old, admitted to any of the pediatric or neonatal ICUs in Manaus, from 2004 to 2009, with parasitological diagnosis of *P. vivax*.

Patient	Age/gender	Time of disease (days)	Associated acute comorbidity	Associated chronic comorbidity	Respiratory distress	Severe anemia	Coma	Jaundice	Shock	Metabolic acidosis	Low glucose	Blackwater fever (ARF)	PIM score (%)	Outcome
1	6 y/M	7			+				+				1.9	Recovered
2	1 mo/M	7			+	+		+		+			24.8	Recovered
3	8 y/F	3	Viral encephalitis*				+		+				45.9	Died
4	1 y/F	4	Gastroenteritis	Malnutrition*	+				+	+			28.1	Recovered
5	3 y/M	4	Rotavirus gastroenteritis*	Cystic fibrosis*	+								1.6	Recovered
6	1 mo/M	3				+		+	+	+			9.1	Recovered
7	1 mo/M	1	Gastroenteritis and drug-induced hepatitis		+	+			+	+			21.1	Recovered
8	1 y/M	2		Neurological sequelae	+ (ARDS)		+		+	+	+		40.5	Recovered
9	2 y/F	25			+ (ARDS)								9.9	Recovered
10	11 y/M	7		Malnutrition	+			+	+	+			24.9	Recovered
11	7 mo/F	26	Rotavirus gastroenteritis+Sepsis (*Acinetobacter* sp.*+Klebsiella* sp.)*		+ (ARDS)				+				11.4	Recovered
12	8 y/M	10		G6PD deficiency*		+						+	4.0	Recovered
13	3 y/M	23			+				+	+			11.3	Recovered
14	3 y/M	7		G6PD deficiency		+					+	+	2.4	Recovered
15	2 y/M	2			+						+		8.4	Recovered
16	2 y/M	4	Bacterial pneumonia with lung empyema*		+				+	+			12.6	Recovered
17	8 mo/F	15			+				+				4.1	Recovered
18	2 mo/F	10	Urosepsis (*Klebsiella* sp.)*	Neurological sequelae		+	+						7.5	Recovered
19	3 y/M	7			+		+		+				55.7	Died
20	1 mo/M	8	Rotavirus gastroenteritis	Malnutrition		+				+			8.7	Recovered
21	2 y/F	3		ITP*									2.0	Recovered
22	1 y/M	31			+		+		+				36.6	Recovered
23	5 mo/M	6	Rotavirus gastroenteritis	Malnutrition	+					+			2.8	Recovered
24	18 d/M	5						+					1.2	Recovered

y years; mo: months; d: days; NA: Non-available; ARDS: Acute Respiratory Distress Syndrome; ARF: Acute Renal Failure; PIM: Pediatric Index of Mortality; ITP: Immune Trombocytopenic Purpura.

* **Viral encephalitis** confirmed by autopsy; diagnosis of **malnutrition** confirmed by body mass index (BMI) Z-score <−2; **rotavirus gastroenteritis** confirmed by immunochromatographic rapid test in the stool; **cystic fibrosis** suggested by lung biopsy; **sepsis** confirmed by two positive blood cultures; **G6PD deficiency** confirmed by qualitative Brewer's test; **lung empyema** confirmed by computed tomography; **urosepsis** confirmed by both positive urine and blood culture; **ITP** confirmed by ASH criteria; ARDS defined as acute bilateral lung infiltrates and a PaO_2_∶FiO_2_<200 mmHg.

**Table 3:** Detailed laboratorial information from 24 children 0–14 years old, at the moment of admission to any of the pediatric or neonatal ICUs in Manaus, from 2004 to 2009, with parasitological diagnosis of *P. vivax*.

Patient	Hb (g/dL)	Leukocyte/mm^3^	Platelets/mm^3^	AST (mg/dL)	ALT (mg/dL)	Creatinin (mg/dL)	Total bilirubin (mg/dL)	Glucose (mg/dL)
1	9.1	18,600	49,000	286	253	0.7	0.9	90
2	2.8	25,600	34,000	160	111	0.6	4.3	55
3	8.5	6,400	161,000	23	15	0.6	0.6	70
4	8.5	19,300	180,000	73	22	1.9	0.3	304
5	10.4	9,000	378,000	20	30	1.0	1.0	199
6	2.5	28,600	13,000	83	48	0.6	8.3	59
7	5.0	22,500	16,000	961	1400	2.7	1.0	64
8	9.5	13,700	113,000	15	23	0.8	1.0	28
9	8.3	30,100	207,000	17	19	0.5	1.0	64
10	8.6	12,700	129,000	23	27	0.8	6.7	88
11	7.9	57,700	93,000	34	45	1.1	1.0	67
12	5.0	12,100	170,000	222	53	7.5	2.7	42
13	5.9	10,100	174,000	56	10	0.4	1.0	63
14	4.2	18,700	303,000	29	26	7.1	1.4	39
15	8.8	4,600	78,000	30	28	0.4	1.0	38
16	6.5	16,600	611,000	25	15	0.9	1.0	75
17	9.1	16,800	196,000	22	29	0.4	1.0	90
18	5.0	31,500	76,000	34	43	0.4	1.0	67
19	7.4	8,500	190,000	133	46	0.3	2.8	98
20	3.3	31,600	19,000	30	45	0.8	1.6	70
21	6.2	29,600	6,000	20	17	0.9	1.0	88
22	8.7	19,600	104,000	159	64	0.7	1.0	52
23	7.3	11,200	69,000	34	50	1.0	1.0	70
24	7.4	2,100	15,000	45	23	1.0	24.2	101

Hb: hemoglobin; ALT: alanine aminotransferase; AST: aspartate aminotrasferase.

Table 4 summarizes the results of the univariate and multivariate logistic regression evaluating risk factors associated with ICU admission. Males [OR = 5.90 (95% CI = 1.28–34.25); (p = 0.039)], age <5 years [OR = 6.36 (95% CI = 2.67–13.63); (p = 0.028)], peripheral parasitemia greater than 500/mm^3^ [OR = 4.94 (95% CI = 1.31–18.63); (p = 0.018)], the presence of acute [OR = 8.13 (95% CI = 1.33–49.81); (p = 0.023)] and/or chronic co-morbidities [OR = 6.02 (95% CI = 1.38–26.35); (p = 0.017)] were all independently associated with the risk of developing life-threatening *P. vivax* monoinfection requiring admission to an ICU. None of the children not admitted to the ICU developed severe disease after their discharge, as reviewed in their electronic files from follow-up visits in the outpatient clinics.

**Table 4:** Univariate and multivariate (logistic regression) analysis of factors associated to the admission in the ICUs of children 0–14 years old with *P. vivax* infection, in Manaus, from 2004 to 2009.

	Cases admitted to the ICU n/N (%)	Patients not requiring admission to the ICU n/N (%)	Crude odds ratio (95% CI)	Adjusted odds ratio (95% CI)	p-value
Male sex	17/24 (70.8)	53/91 (58.2)	1.91 (0.92–5.71)	5.90 (1.28–34.25)	0.039
Age (<5 years)	20/24 (83.3)	25/91 (27.5)	4.64 (2.05–7.65)	6.36 (2.67–13.63)	0.028
Peripheral parasitemia (>500 parasites/mm^3^)	14/19 (73.6)	32/89 (36.0)	4.99 (1.65–15.12)	4.94 (1.31–18.63)	0.018
Time of acute disease prior to treatment (>7 days)	8/24 (33.3)	22/91 (24.2)	1.57 (0.60–4.16)	3.39 (0.81–14.10)	0.093
Presence of any chronic disease	10/24 (41.7)	9/91 (9.9)	6.50 (2.25–18.86)	6.02 (1.38–26.35)	0.017
Presence of any other acute co-morbidity	9/24 (37.5)	7/91 (7.7)	7.20 (2.33–22.30)	8.13 (1.33–49.81)	0.023

Table 5 compares ICU admitted *P. vivax* monoinfections with hospitalized *P. vivax* patients not requiring ICU admission. Of all variables assessed for both groups, the odds of being admitted to the ICU increased in patients fulfilling at least one WHO severity criterion, particularly in those with severe anemia or coma. The mean of the PIM, a validated score that parallels bad prognosis in pediatric ICUs, was higher in children with shock and coma. *P. falciparum* or *P. falciparum/P. vivax* infected children (30.0%) died more frequently than those infected with *P. vivax* monoinfection (8.3%), but this difference was not significant (Yate's corrected Fisher's exact test; p = 0.138). *P. falciparum* monoinfection showed higher fatality (2/4; 50.0%) when compared to *P. vivax* monoinfection (2/24; 8.6%), but this difference likewise was not significant (p = 0.086).

**Table 5:** Descriptive data and univariate analysis of *P. vivax* monoinfection cases admitted to the ICU versus patients not requiring ICU admission, according to the WHO type of severity.

	Cases admitted to the ICU n/N (%)	Patients not requiring ICU admission n/N (%)	Odds ratio (95% CI)	p-value	PIM Score (%) of cases (Mean ± SD)
WHO criteria for malaria severity	23/24 (95.8)	17/91 (18.7)	100.11 (12.60–793.60)	<0.001	16.2±15.6
Respiratory distress	16/24 (66.7)	0/91 (0.0)	-	-	18.4±15.7
Shock (algid malaria)	13/24 (54.2)	0/91 (0.0)	-	-	23.3±17.0
Metabolic acidosis	10/24 (41.6)	-	-	-	16.0±12.3
Severe anemia	7/24 (29.2)	5/91 (5.5)	7.08 (2.00–24.97)	0.003	11.0±8.5
Coma	5/24 (20.8)	2/91 (2.2)	11.71 (2.11–64.94)	<0.001	37.2±18.1
Hyperbilirrubinemia	4/24 (16.7)	11/91 (12.1)	1.45 (0.42–5.05)	0.513	15.0±11.8
Hypoglycemia	3/24 (12.5)	0/91 (0.0)	-	-	17.1±20.4
Acute renal failure (ARF) due to hemoglobinuria	2/24 (8.3)	0/91 (0.0)	-	-	3.2±1.1
Death	2/24 (8.3)	0/91 (0.0)	-	-	50.8±6.9

## Discussion

This study showed that in Manaus most of the malaria-associated hospitalizations in ICUs were related to *P. vivax* during the period of study, confirming the public health impact of this infection [19]–[20]. Male sex, age less than five years, parasitemia greater than 500/mm^3^, and the presence of any acute or chronic co-morbidity were independently associated with ICU admission. At least one of the WHO severity criteria for malaria (formerly validated for *P. falciparum* infection only) was present in most of the patients admitted to the ICU. WHO severity criteria may also serve as predictors of severity for *P. vivax* patients. Further studies, however, are needed to confirm this finding considering the small number of cases presented in our series.

A significant male sex predominance among children admitted to the ICU in our study contrasts with data from Indonesia and India, where the female sex was associated with severity in vivax infection [3], [21]. One possible explanation of this difference is the heterogeneous age range of infected children in these continents.

Similar to what was observed by Tjitra and colleagues in Papua, Indonesia [3], respiratory distress, present in two-thirds of patients in our series, was the most frequent complication among patients admitted to the ICU with *P. vivax* infection, and had a particularly poor prognosis. In *P. falciparum* malaria, respiratory distress arises secondary to either primary pulmonary pathology mediated by sequestration of parasitized erythrocytes in the lung microvasculature or from the drive to compensate metabolic acidosis. For *P. vivax* malaria, however, the pathophysiology behind respiratory distress is not well understood. Studies performed in uncomplicated vivax patients suggest that progressive alveolar-capillary dysfunction occurs after treatment, and this inflammatory response is proportional to initial parasite burden in *P. vivax* [22]. More recently, *ex vivo* adhesion of *P. vivax*-parasitized red blood cells to human lung endothelial cells was demonstrated for the first time [23], suggesting that cytoadhesion could play a role in the pathogenesis of this disease. Although, in a patient who died of ARDS, histopathological analysis did not show any evidence of cythoadhesion [24]. Patient outcomes in this series support the hypothesis of a primary pulmonary lesion driving the respiratory clinical manifestations, complicated by the presence of concomitant metabolic acidosis or severe anemia. Half of the 16 patients with respiratory distress had concomitant metabolic acidosis, but only 2 had severe anemia simultaneously. Pneumonia is another confounding diagnosis of respiratory distress, and in all the children in the present report antibiotics were prescribed empirically.

The occurrence of severe anemia also paralleled data from other endemic areas [25], and was consistent with previous data published from Manaus [11], [26]. Further corroborating data from Indonesia [27], we observed that 5 out of 6 children under 3 months of age presented severe anemia, confirming *P. vivax* as a cause of severe morbidity in the early infancy. Severe anemia can also be seen, not triggered by the parasite itself, in patients with G6PD deficiency, as a complication of the use of primaquine for the radical cure of hypnozoites, as observed in three patients described here (two with pure *P. vivax* and one with mixed infection). If followed by acute renal failure (ARF), it constitutes the syndromic presentation of blackwater fever [28]. Acute renal failure was mostly related to acute hemolysis in patients with G6PD deficiency. The possibility of concomitant *P. malariae* infection, a species known to cause acute or delayed renal complications seems highly improbable, as this species is not detected in Manaus [33]. G6PD deficiency seems to be frequent in Brazilian malaria-endemic areas, where it is not routinely screened before the prescription of antimalarials. The prevalence of the A^−^ variant among males has been estimated at 3% [29], and a number of hospitalizations due to this complication have been reported [30]. If G6PD deficiency is not ruled out in epidemiological studies focusing on vivax-related anemia, this complication triggered by the parasite per se could be falsely overestimated.

Circulatory collapse (algid malaria) associated with *P. vivax* infection was both frequent and severe, requiring the use of vasopressors, with or without a confirmed positive blood culture for aerobes. Even for *P. falciparum* malaria, the etiology of this complication is uncertain and the potential role of septic shock as a concomitant entity has been proposed [31]. In such cases therefore, blood cultures are mandatory to rule out bacterial co-infections, which may be related to severity and consequently to ICU hospitalization.

The presence of hyperbilirubinemia is becoming recognized recently as an inappropriate criterion of severe disease if found in isolation [32]. In our data this laboratory finding was not predictive of ICU hospitalization [OR = 1,45 (CI = 0,42–5,05), p = 0,513]. Jaundice detected on a routine physical exam, however, could still be used as a warning sign of severity, given that in 3/4 cases jaundice was accompanied by another severity criterion. As serology for the common viral hepatitis in this region was not requested, it is difficult to ascertain whether such episodes were primarily caused by *P. vivax per se* or resulted from a concomitant liver infection.

Coma was also reported in our series, but in 3 out of 5 children with coma, additional explanations other than cerebral malaria could be found, such as viral encephalitis in one, and decompensation of previous neurological sequelae in two. In one patient hypoglycemia could also be a superimposed factor. Cerebral vivax malaria actually seems to be more common in children [34], but the possibility of other infectious diseases of the central nervous system has to be taken into consideration given the low prevalence of cerebral malaria in vivax infection [35].

It is noteworthy that acute or chronic co-morbidities were detected in 58% (14/24) of the patients with *P. vivax*. This was also observed in an almost identical proportion (6/10) of *P. falciparum* patients, which leads to the conclusion that the presence of co-morbidities may contribute to or facilitate the appearance of complications in all malaria. Importantly, this aspect seems to have been specially underreported in prospective studies on vivax severity [3]. Recent data suggests that *P.vivax*-triggered anemia could be related to parvovirus B19 infection [36]. In our series, rotavirus infection was found among ICU-admitted *P. vivax* (4 cases) and *P. falciparum* (2 cases) children. Despite the lack of local data on rotavirus infection, the worldwide-recognized increased severity in those less than 5 years of age could in part explain the younger age of the ICU hospitalized children. Furthermore, in our children, septic shock with confirmed bacteremia was observed in three cases. In Mozambique, the systematic performance of blood cultures on hospital admissions showed that a positive culture for aerobes in children with malaria was associated with an increased risk of death [37]. Therefore, a predisposition to malarial infection in the setting of other bacterial infections may apply to all *Plasmodium* species. The finding of four malnourished children among vivax cases admitted to the ICU does not seem negligible, and should be taken into consideration as a potential confounding factor when severity frequencies are compared across the world [38]. Although *P. vivax* can surely independently cause severe or even life threatening episodes in adults and children, the relative importance of co-morbidities or other chronic existing conditions in accelerating the progression to a severe disease cannot be disregarded.

Despite the WHO recommendation to treat patients with severe *P. vivax* malaria as patients with severe *P. falciparum/P. vivax* malaria, due to the possibility of submicroscopic parasitemia of the first species, the Brazilian Guidelines for Antimalarial Treatment have only recently changed. During the study period of the present study, chloroquine was prescribed to all patients. Resistance to chloroquine was described for the first time in Latin America in Manaus [39], and more recent data suggest that *in vivo* resistance in this locality is estimated at 10% [40]. However the resistance does not seem to be disseminated throughout the country [41], and the first line treatment for *P. vivax* is still chloroquine. Ecological studies suggest that severity in vivax infection could be related to chloroquine-resistance [42]. These and previous data, however, show a good response to chloroquine in severe patients, and suggest that in the Brazilian Amazon this association is not well understood [11].

The fatality rate among *P. vivax* patients hospitalized in the ICU (8.3%) was not insignificant, even in the Brazilian scenario where diagnosis and treatment of malaria are free and universally available. These numbers could be far larger, however, if a reasonably established health system with tertiary care facilities is not in place. Our small sample was unable to show that *P. falciparum* children died more frequently than those infected with *P. vivax*. Actually data from other settings point to a similar fatality rate between the two species [21], [43]. From our data it is possible to detect a high risk of ICU admission among children with mixed infections, however this finding needs to be confirmed in other similar settings in Latin America. PIM score predicted mortality relatively well and its use should be emphasized in future similar pediatric studies.

An important limitation of this study is its retrospective design, and all analyzed information is based on the available registration in clinical charts. Besides that, the absence of PCR confirmation of malarial infections is an additional limitation. Species-specific PCR increases the diagnostic accuracy and can detect submicroscopic infections that may have been disregarded only after standard microscopy. It is possible therefore, that some of our assumed *P. vivax* monoinfections may in fact be mixed cases. Although possible, this seems unlikely, as a preliminary analysis from our laboratory shows that only 5% of the positive TBSs for *P. vivax* identify mixed infections, based on real-time PCR (data not shown). Another limitation is the lack of information on total number of patients hospitalized due to malaria in Manaus in the period.

In the present study we showed that, in absolute numbers, *P. vivax* infection was responsible for most of the ICU admissions of children 0–14 years old due to severe malaria (despite of similar proportional numbers when compared to *P. falciparum* infections reported), and that the clinical complications were very similar to those seen in *P. falciparum* in this same endemic area. Despite the fact that peripheral parasitemia greater than 500/mm^3^ was associated with ICU hospitalization, the positive predictive value of this cut-off for predicting need for ICU admission is extremely low, given the tiny fraction of malaria cases requiring ICU in the general population. Severe vivax patients should also trigger the routine screening of additional infectious diseases in the daily practice in the tropics. From the public health perspective, decision-makers from national malaria programs should re-orientate clinical management algorithms in order to improve treatment, diminish the burden of hospitalization, decrease costs and reduce malaria-associated mortality.

From the basic science perspective, pathogenesis studies in severe vivax should carefully rule out other diseases, which may alter the immune response [44]. Definitely respiratory distress, shock and anemia are major complications associated with *P. vivax* infection, for which mechanisms are poorly understood even in the case of severe *P. falciparum*. More studies are needed to evaluate the applicability of these findings in other age groups. Due to the fact that studies on the clinical aspects and biology of *P. vivax* have been neglected during most of the 20^th^ century, the large knowledge gaps regarding this parasite [45] may pose challenges for future malaria eradication plans [46].
